# Epigenetic Modification of PD-1/PD-L1-Mediated Cancer Immunotherapy against Melanoma

**DOI:** 10.3390/ijms23031119

**Published:** 2022-01-20

**Authors:** Hikaru Nanamori, Yu Sawada

**Affiliations:** Department of Dermatology, University of Occupational and Environmental Health, 1-1 Iseigaoka, Yahatanishi-Ku, Kitakyushu, Fukuoka 807-8555, Japan; hikaru-n@med.uoeh-u.ac.jp

**Keywords:** malignant melanoma, anti-PD1 antibody, anti-PD-L1 antibody, epigenetics

## Abstract

Malignant melanoma is one of the representative skin cancers with unfavorable clinical behavior. Immunotherapy is currently used for the treatment, and it dramatically improves clinical outcomes in patients with advanced malignant melanoma. On the other hand, not all these patients can obtain therapeutic efficacy. To overcome this limitation of current immunotherapy, epigenetic modification is a highlighted issue for clinicians. Epigenetic modification is involved in various physiological and pathological conditions in the skin. Recent studies identified that skin cancer, especially malignant melanoma, has advantages in tumor development, indicating that epigenetic manipulation for regulation of gene expression in the tumor can be expected to result in additional therapeutic efficacy during immunotherapy. In this review, we focus on the detailed molecular mechanism of epigenetic modification in immunotherapy, especially anti-PD-1/PD-L1 antibody treatment for malignant melanoma.

## 1. Introduction

The skin has a complex three-dimensional structure containing various component cells and is an organ located in the outmost layer of the human body. The skin is exposed to various influences from environmental alterations and stimuli, such as temperature, chemicals, microorganisms, and medications [1,2,3,4,5,6,7,8,9,10]. Indeed, representative inflammatory skin diseases such as psoriasis and atopic dermatitis are significantly influenced by various environmental factors [11,12,13,14,15]. Because of its characteristics as an outer organ, the skin is developed to tolerate, and is specialized to adapt to, these environmental changes [16]. As one of the explanations of this flexibility to these environmental factors, the epigenetic alteration mechanism in the skin is well established.

Recent advancements in the knowledge gained from research demonstrate the importance of epigenetic modification in the pathogenetic role of skin cancers [17,18,19]. The tumor obtains advantages in development via epigenetic modification. In addition, these epigenetic changes in the tumor drive the escape phenomenon in anti-tumor immune responses [20,21]. However, there are a limited number of studies regarding the detailed molecular mechanisms of epigenetic changes in both host immunity and malignant melanoma.

Immunotherapy is currently developed for various tumors, and PD-1/PD-L1 targeted therapy is especially widely used for advanced or metastatic malignancies, showing a high therapeutic efficacy [22,23,24]. PD-1 is expressed on the surface of T cells following activation and negatively regulates inflammation in infections and cancer [25,26]. T-cell proliferation, cytokine production, and cytolytic activity are inhibited following PD-L1 binding to PD-1, leading to functional inactivation of T cells [27]. The current problem is that not all patients with malignant melanoma can obtain therapeutic efficacy [28,29]; therefore, it is desirable to develop some additional therapeutic options for the enhancement of the efficacy of immunotherapy.

The aim of this study is to explore the future therapeutic candidate options for epigenetic modifiers for combination with immunotherapy in malignant melanoma. To cover the unknown mechanism underlying epigenetic changes in malignant melanoma, we also explored the epigenetic changes in other tumors, to obtain a better understanding of the epigenetic influence in PD-1/PD-L1-targeted immunotherapy.

## 2. Epigenetic Modification

**Figure 1:**
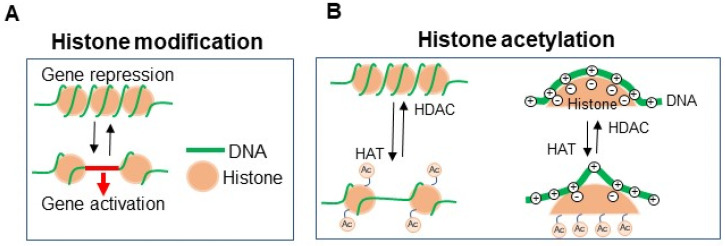
Epigenetic histone modification (**A**) Histone modification. DNA binds to histone for gene repression, while the weak connection of DNA and histone activates gene transcription. (**B**) Histone binds to DNA via voltage connection, which is canceled by HAT-mediated histone acetylation. HDAC cancels the histone acetylation, leading to gene repression.

The majority of DNA sequencing information does not change throughout life; however, the manipulation of gene expression is possibly mediated by the chemical modification of DNA itself or by DNA-binding proteins such as histone [30,31,32,33,34,35] (Figure 1A). This epigenetic modification can alter the function of the skin, and it is possible for the skin to adapt to these environmental changes [16,36]. In addition, epigenetic modification in the skin influences various inflammatory skin diseases and cancers [37,38,39,40]. For these reasons, the skin is recognized as one of the organs most influenced by the environments outside the human body. In this section, we introduce representative epigenetic modifications associated with immunotherapy against melanoma, to obtain a better understanding of the detailed molecular mechanisms of epigenetic-modification-targeted therapy, which is discussed in the following section.

### 2.1. DNA Methylation

The CqG island is often influenced by DNA methylation because this site enriches DNA regions with a cytosine nucleotide followed by a guanine nucleotide in a linear sequence from a 5′ to 3′ direction. CqG islands are often observed in gene promoter sites. DNA methylation basically silences targeted gene expression.

### 2.2. Histone Methylation

Histone methylation primarily targets histone H3 lysine residue and induces both activation and suppression of gene transcription. Histone methyltransferase enhances the methylation of histone, while histone demethylase cancels the methylation of histone.

### 2.3. Histone Acetylation

Histone acetylation targets the lysine residue of histone. DNA and histone bind to each other via a voltage charge connection, and histone acetylation reduces the positive charge in histone, resulting in a weakening of the voltage connection with DNA, leading to the activation of gene transcription. Histone acetyltransferase (HAT) accelerates histone acetylation, leading to the enhancement of gene transcription. In contrast, histone deacetylase (HDAC) suppresses the acetylation of histone and represses gene transcription (Figure 1B).

### 2.4. Histone Ubiquitination

Ubiquitination targets histone H2A and H2B, alters the chromatin structure, and enhances the access of other enzymes involved in gene transcription. This epigenetic modification occurs in both gene transcriptional activation and repression.

## 3. The Characteristics of Malignant Melanoma

Malignant melanoma is recognized as one of the most lethal skin cancers, derived from the malignant transformation of melanocytes [41]. Melanoma is commonly observed in the skin and rarely recognized in pigment-producing cells located in other sites, such as the gastrointestinal tract, eye, and genitals [42,43,44]. Although the incidence rate is different in each country, the incidence of malignant melanoma is gradually increasing across the world [45,46]. Ultraviolet light exposure is one of the risk factors for malignant melanoma. UV radiation dysregulates the tumor suppressor gene p53, which contributes to the apoptosis of tumor cells [47].

Therefore, malignant melanoma is a representative highlighted skin cancer for clinicians to study with respect to the pathogenesis and therapeutic strategy. The therapeutic strategy is based on the clinical stages according to the TNM classification [48].

In the past, the traditional therapeutic option dacarbazine was used for the treatment of advanced-stage malignant melanoma; however, its efficacy could not achieve a satisfactory level with respect to clinical outcomes [49]. However, this undesirable situation completely changed with the development of anti-PD-1/PD-L1 antibody treatment. Anti-PD-1 antibody treatment dramatically improved the prognosis for advanced malignant melanoma [50,51,52]. In addition, BRAF inhibitor plus MEK inhibitor combination therapy is also available for malignant melanoma patients with positive BRAF mutation (BRAFV600E/K mutation) [53,54]. Several gene mutations are known to be associated with malignant melanoma [55]. One of the representative mutations is the BRAF gene, which is involved in cell growth and apoptosis mediated by the mitogen-activated protein kinase (MAPK) activation [56]. The BRAF V600 mutation is observed in approximately 50% of malignant melanomas [57].

PD-1 is a type I transmembrane glycoprotein. The PD-1 cytoplasmic domain induces negative cellular signals through its immunoreceptor tyrosine-based inhibitory motif (ITIM) and immunoreceptor tyrosine-based switch motif (ITSM) [58]. PD-1-deficient mice developed lupus-like glomerulonephritis [59]. Furthermore, anti-PD-1 antibody administration leads to autoimmune adverse events such as thyroiditis [60]. Therefore, PD-1 prevents excessive immune reactions.

Currently, PD-1 and CTLA-4 are the therapeutic targets against malignant melanoma, and anti-PD-1 antibody (nivolumab) and anti-CTLA-4 antibody (ipilimumab) are currently used as single-agent or combination therapies for advanced or recurrent cases, as well as adjuvant therapy after surgery. The overall one-year survival rate was better in patients treated with nivolumab (72.9%) than in those treated with dacarbazine (42.1%) [49]. Recent studies revealed that the efficacy of immunotherapy seems to depend on the clinical subtypes of malignant melanoma. Acral melanoma, especially, showed a lower efficacy of immunotherapy [61]. One of the reasons is that acral melanoma exhibits a lower mutation burden, possibly leading to lower recognition of the antigen by host immune cells [62]. Since all cases could not obtain therapeutic efficacy using these immunotherapies [28,29], an additional therapeutic combination option with immunotherapy is currently desired for the treatment of malignant melanoma.

## 4. Epigenetic Modification and Potent Therapeutic Efficacy of the Combination with Anti-PD-1/PD-L1 Antibody Treatment

In this section, we summarize the detailed molecular mechanisms of epigenetic modification to clarify the possible beneficial impact on enhancing the efficacy of immunotherapy. In addition, therapeutic outcomes of the combination therapy with anti-PD-1/PD-L1 antibody treatment are also discussed.

## 5. HDAC Inhibitor Plus Anti-PD-1 Antibody Improves Survival

HDAC inhibitors have the potential to enhance anti-tumor immune reactions. Type I HDAC inhibitors such as panobinostat, entinostat, and mocetinostat can enhance PD-L1 expression in melanoma both in vivo and in vitro. HDAC inhibitors show a synergy effect with anti-PD-1 antibody treatment, giving an improved survival rate in a melanoma mouse experiment [63]. In other tumors, the combination treatment of entinostat and anti-PD-1 antibody also improved survival in both a breast cancer mouse model and a metastatic pancreatic cancer mouse model [64], suggesting that these combination therapies might be useful for the treatment of various malignancies.

The efficacy of immunotherapy against persistent skin melanoma or uveal melanoma is enhanced by HDAC inhibitor [65]. Entinostat upregulates HLA expression and PD-L1 in the tumor [65]. A melanoma mouse model bearing B16-F10 melanoma cells treated with PD-1 showed a moderate therapeutic efficacy, which could be upregulated by entinostat [65].

A phase 2 clinical trial was conducted to clarify the efficacy of entinostat combined with pembrolizumab in patients with metastatic uveal melanoma [66]. The objective response rate was obtained in 14% of patients with metastatic uveal melanoma [66]. The median progression-free survival was 2.1 months, and the median overall survival was 13.4 months [66]. Therefore, the combination therapy with HDAC inhibition and anti-PD1 immunotherapy is expected to obtain better therapeutic outcomes in patients with metastatic uveal melanoma [66].

In contrast, other malignancies did not show the same therapeutic benefit with the combination of HDAC inhibitor and anti-PD-1 antibody treatment. In a murine hepatocellular carcinoma model, an HDAC inhibitor, belinostat, activated the antitumor action against hepatocellular carcinoma in combination with anti-CTLA-4, while anti-PD-1 therapy could not obtain enough additive effect of the HDAC inhibitor against hepatocellular carcinoma [67]. Another HDAC inhibitor, CG-745, showed a synergy effect with anti-PD-1 antibody treatment [68]. Therefore, the therapeutic effect might depend on the affinity of the HDAC inhibitor under the tumor-specific microenvironment. The actual impact of the HDAC inhibitor with anti-PD-1 antibody treatment is required to be verified by further clinical trials.

## 6. HDAC Inhibitor Plus Anti-PD-1 Antibody Enhances Cytotoxic Reaction

Cytotoxic immune action is a representative anti-tumor immune response mediated by CD8+ cells and NK cells. The combination therapy of entinostat and anti-PD-1 antibody activates the functioning of cytotoxic CD8+ effector T cells to decrease tumor size [64]. Vorinostat or entinostat also enhances NK-cell-mediated tumor cell lysis [69]. A histone deacetylase inhibitor, CG-745, also upregulates the anti-tumor action of anti-PD-1 antibody [68]. CG-745 activates IFN-γ expression, and cytotoxic T cell and NK cell proliferation [68]. CG-745 also impairs Treg proliferation and M2 macrophage polarization [68]. An HDAC inhibitor treatment with chidamide alone enhances cytotoxic immune cell reaction in triple-negative breast cancer [70].

HDAC inhibitor reactivates exhausted T cells via the alteration of the epigenetic modification. A diethylenetriamine–vorinostat encapsulated siRNA-PD-L1 drug delivery system was developed [71], and the vorinostat-loaded vesicle exhibited a high efficacy in inducing a cytotoxic reaction and apoptosis in the tumor cells in vivo [71].

Granzyme B is reversible with respect to the anti-tumor cytotoxic effect exerted by HDAC inhibitors [72]. Granzyme B is a serine protease that initiates cell apoptosis mediated by activation of various caspases such as caspases 3 and 7, leading to DNA disruption [73]. Therefore, these cytotoxic molecules are essential for the anti-tumor immune response to cancers [74,75] and are expected to be manipulated by the treatment with HDAC inhibitors.

## 7. HDAC Inhibitor Enhances PD-L1 Expression in Tumors

HDAC inhibitor activates PD-L1 expression in tumor cells both in vitro and in vivo [69]. These findings suggest that the HDAC inhibitor might obtain anti-tumor efficacy in combination with PD-L1 inhibitor treatment. HDAC inhibitor also enhances other tumors’ PD-L1 expression. Chidamide upregulates the expression level of PD-L1 in cancer cells of triple-negative breast cancer, contributing to T-cell recognition and PD-1/PD-L1 blockade therapy response [70].

As the mechanism, chidamide could regulate PD-L1 transcription by affecting the transcription factor STAT1 that binds to the promoter site of PD-L1 [76]. This HDAC inhibitor effect seems to be a disadvantage for the anti-tumor immune response; therefore, a single HDAC inhibitor treatment might not obtain enough therapeutic potency against cancers, as shown in previous studies [77].

## 8. HDAC Inhibitor Enhances HLA Class I/MHC Class I Expression in Tumors

The combination of panobinostat with anti-PD-1/PD-L1 antibody enhances HLA class I surface expression in Merkel cell carcinoma and the infiltration of CD8+ T cells into Merkel cell carcinoma tumor tissue [78]. As one of the refractory mechanisms of PD-1/PD-L1 treatment, MHC class I was downregulated. HDAC inhibitors are expected to improve this disadvantage of anti-PD-1/PD-L1 antibody treatment through the downregulation of HLA class I during anti-PD-1/PD-L1 antibody treatment against Merkel cell carcinoma [78]. Additionally, chidamide upregulates the expression level of MHC class I and II in tumor cells of triple-negative breast cancer [70]. Although the detailed mechanism is still unknown, these findings suggest that anti-PD-1/PD-L1 antibodies against melanoma cells might also impair HLA class I/MHC class I expression, which might be canceled by HDAC inhibitors.

## 9. HDAC Inhibitor Enhances MHC Class II in Tumors

MHC class II is a vital immune component that enhances the adaptive immune response [79]. MHC class II expression is limited on the surface of antigen presentation cells, and tumor cells also express MHC class II, which is closely related to the lymphocyte infiltration around the tumor [80]. The HDAC inhibitor upregulates MHC class II expression in non-small-cell lung cancer, suggesting the efficacy of combination therapy with anti-PD-1 antibody treatment [81]. In particular, MHC class II plays a crucial role in antigen presentation to naïve T cells for the induction of cytotoxic T cells [82]. Therefore, HDAC inhibitor treatment is expected to show the booster effect in combination with immunotherapy.

## 10. HDAC6 Enhances Anti-Tumor Effects

HDACs expressions were closely associated with the prognosis in lung cancer in a mouse experiment [83]. Low HDAC6 expression was closely associated with favorable clinical behavior [83]. A selective HDAC6 inhibitor decreases IL-1β and IL-6 production and PD-L1 expression in tumor cells [83]. The combination therapy with anti-PD-1 antibodies activates cytotoxic CD8+ T-cell induction and decreases the tumor sizes [83]. Consistently, the adoptive transfer of HDAC6-deficient CD8+ T cells to Rag1-deficient mice impairs cytotoxic CD8+ T-cell responses against vaccinia infection and impairs perforin expression in cytotoxic T cells [84], suggesting that the anti-tumor immune effect of HDAC6 is possibly mediated by the upregulation of cytotoxic function.

## 11. HDAC3 Inhibitor for Upregulation of PD-L1 Expression

HDAC3 is a class I HDAC and plays a vital role in the regulation of gene transcription in cooperation with nuclear repressor complexes containing nuclear receptor corepressor (NCOR) or silencing mediator of retinoic and thyroid receptors (SMRT) corepressors [85,86,87]. HDAC3 suppresses PD-L1 transcription in B-cell lymphoma. HDAC3 inhibitor enhances histone acetylation and recruitment of BRD4 at the promoter region of the PD-L1 gene, leading to transcriptional activation in B-cell lymphoma [88]. In addition, HDAC3 suppresses DNMT1, which is a positive driver of PD-L1 transcription. HDAC3 inhibition can also enhance PD-L1 expression in DC, and the combination therapy with anti-PD-L1 antibody enhances anti-tumor effects [88]. Although HDAC3 inhibition can enhance PD-L1 in the tumor, PD-L1 in dendritic cells is also suppressed. Therefore, the combination therapy of HDAC3 inhibitor plus PD-1/PD-L1-targeted therapy is also expected to obtain therapeutic efficacy. In addition, HDAC3 inhibition might show cytotoxic tumor effects. HDAC3-deficient CD8+ cells enhance granzyme B expression and anti-viral effects in a chronic viral infection model [89], suggesting that HDAC3-targeted inhibition is a potent therapeutic treatment against cancers via enhancement of cytotoxic molecule actions against cancer.

## 12. DNMT and HDAC6 Combination Inhibitor Treatment Enhances Cytotoxic Immune Reaction in Ovarian Cancer

DNMT inhibitor treatment might also be desired for future epigenetics-targeted therapies for malignant melanoma. An animal model of ovarian cancer shows an increased cytotoxic reaction to tumor cells treated with a combination of HDAC6 and DNMT inhibitors via enhancement of the function of IFN-g-producing CD8+ cells, NK cells, and NKT cells, leading to improvement in survival [90]. Although there is no study regarding the combination with anti-PD-1/PD-L1 antibody, triple therapy of HDAC6 and DNMT inhibitors and anti-PD-1/PD-L1 antibody might show a high therapeutic effect against melanoma.

## 13. DNA Methylation Upregulates PD-1 and Decreases PD-L1/L2

Tumor carriers epigenetically modulate PD-1 expression, possibly by obtaining an advantage for the immune escape phenomenon. Hepatocellular carcinoma patients’ PBMC showed DNA methylation in T cells during early-stage hepatocellular carcinoma derived from chronic hepatitis B and C. DNA methylation is associated with highly enriched immune-function-associated genes such as PD-1 [91]. PD-1 methylation is identified as a strong prognostic factor in diffuse low-grade glioma patients [92]. Therefore, DNA methylation inhibitor treatment might be a therapeutic strategy for obtaining efficacy against melanoma by suppression of PD-1 expression in immune cells.

Furthermore, 5hmC is an oxidative product in the process of active demethylation of 5mC mediated by the three ten-eleven translocation (TET) enzymes through the oxidative conversion of 5-methylcytosine and 5hmC during cytosine demethylation [93,94]. Therefore, high 5hmC reflects demethylation of the targeted gene, which impairs transcriptional gene suppression. A low content of 5hmC at the PD-1 promoter is observed in effector T cells and depends on the decreased expression of TET [95]. This finding supports the evidence that DNA hypermethylation at the PD-1 promoter site leads to the downregulation of PD-1 expression.

In contrast, DNA methylation decreases PD-L1 expression in colorectal cancer cells. A DNA methyltransferase inhibitor, azacytidine, impairs oxymatrine-induced PD-L1 downregulation in IFN-γ-treated colorectal cancer cells [96]. Therefore, DNA-methyltransferase-targeted inhibitor alone might show a disadvantage in reducing the immunological anti-tumor effect. PD-L2 is designated CD273 and is one of the ligands for PD-1 [97]. PD-L2 acts as a negative regulator for the adaptive immune response [98]. Therefore, PD-L2-targeted treatment is one of the highlighted issues for cancer immunotherapy. Hypermethylation in PD-L2 is closely related to the downregulation of PD-L2 and the enhancement of the infiltration of CD8+ cells, leading to improvement in the survival of patients with gastric adenocarcinoma [99]. DNA hypomethylation leads to the upregulation of PD-L2, which reflects favorable clinical behavior with longer progression-free survival during anti-PD-1 antibody treatment [100]. Although DNA hypermethylation inhibitor enhances PD-L1/PD-L2 expression, it also shows other anti-tumor effects. However, combination therapy with anti-PD-1/PD-L1 antibody might mitigate this disadvantage of DNA methyltransferase inhibitor.

As for other therapeutic options, 5-aza-2-deoxycytidine (DAC) is also clinically available and is used for the treatment of malignancies such as myelodysplastic syndrome [101]. Furthermore, combination therapy with anti-PD-1 antibody treatment showed favorable clinical outcomes in lymphomas [102], suggesting its efficacy for melanoma.

## 14. Lysine-Specific Histone Demethylase 1A (LSD1) Negatively Regulates Anti-Tumor Immune Response

LSD1 is also known as lysine-(K)-specific demethylase 1A (KDM1A) and is a representative histone demethylase [103,104,105]. This histone demethylase promotes demethylation of lysine residues, targeting histone 3, and lysines 4 and 9 (H3K4 and H3K9). LSD1-deficiency activates T-cell infiltration around the tumor and promotes the anti-tumor effects of anti-PD-1 antibody treatment in a mouse melanoma model [106]. Consistently, LSD1 expression shows a negative correlation with CD8+ cell infiltration [106], suggesting that LSD1 inhibitor treatment might become a therapeutic candidate for the immunotherapy of malignant melanoma.

LSD1 is negatively associated with the expression of PD-L1, in addition to the T-cell-attracting chemokines CCL5, CXCL9, and CXCL10 [107]. The combination of LSD1 inhibitor plus anti-PD-1 antibody inhibits tumor growth and pulmonary metastasis via the enhancement of CD8+ T-cell infiltration [107]. LSD1 inhibitor reactivates these chemokines, mediated by increased H3K4me2 at proximal promoter regions [107].

LSD1 deficiency in tumor cells enhances the expression of TGF-β, which plays a vital role in inhibitory effects on cytotoxic CD8+ T cells and subsequently impairs the anti-tumor effect [108]. LSD1 and TGF-β inhibitors in combination with anti-PD-1 antibody treatment enhance CD8+ cell infiltration and cytotoxic reaction [108].

Exhausted CD8+ T cells are recognized in patients with malignant tumors. Therefore, the way to improve these exhausted CD8+ cells is a highlighted target during immunotherapy. LSD1 is associated with these exhausted CD8+ T cells [20]. LSD1 increases exhausted CD8+ T cells, which determine the anti-tumor cytotoxic reaction during anti-PD1 therapy [20].

Several LSD1 inhibitors such as TCP are currently available as clinical cancer therapies [109]. Although the detailed actions against melanoma remain unclear, these chemicals are expected to show an enhancement of anti-tumor immune responses against melanoma.

## 15. DNA Hypermethylation in Pore-Forming Protein Perforin (PRF1)

PRF1 is a membrane-attack-complex/PRF (MACPF) protein family and plays an essential role in the functioning of active cytotoxic T cells and NK cells [110]. DNA methylation in tissue-resident memory cells is associated with their function. DNA methylation in PRF1 in tissue-resident memory cells was observed in 32.9% of urinary bladder cancer patients. DNA hypermethylation in PRF1 is associated with increased PD-1 expression in tumor tissue-resident memory cells, indicating the exhaustion of the immune function [111]. The detailed molecular mechanism of hypermethylation in PRF1 for the enhancement of PD-1 remains unclear. As another role of PRF1 in anti-tumor immune response, PRF1 also maintains the expression of granzyme B in cytotoxic cells [112]. Therefore, DNA hypermethylation inhibitors are also expected to show an enhancement of the cytotoxic reaction in addition to the impairment of PD-1 expression.

## 16. EZH2 Reduces PD-1 Expression

EZH2 is a histone H3 lysine 27 methyltransferase with a component of Polycomb repressive complex 2 (PRC2), and it acts as a downregulator for various targeted genes [113,114,115]. EZH2 is highly upregulated in various malignancies such as melanoma and plays a vital role in cell growth and proliferation; therefore, EH2 is highlighted as an anti-tumor treatment [116,117,118]. EZH2 plays a role in the anti-tumor response of macrophages against mesothelioma cells [119]. A EZH2 selective inhibitor reduces the cytotoxic activity of phagocytosis and induces PD-1 overexpression in macrophages, leading to the development of tumor growth [119].

GSK126 is an EZH2 inhibitor with a potent blood–brain barrier permeability [120]. The combination of this EZH2 inhibitor and anti-PD-1 treatment showed an enhanced anti-tumor effect in syngeneic mouse models. This combination therapy activates T-cell infiltration and reduces tumor growth in murine models of GBM. GSK126 enhances IFN-γ production and subsequently promotes CXCL9 and CXCL10 secretion by the tumor cells, leading to the migration of T cells around tumors [120].

The pharmacological inhibitors directory targets tumor cells and indirectly affects the tumor microenvironment, especially immune cells. Because EZH2 inhibitors reduce immune cell action in some parts, combination therapy with anti-PD-1/PD-L1 antibody might obtain enough therapeutic potency for the EZH2 inhibitor. Tazemezostat is currently undergoing clinical trials for various cancers such as lymphoma [Morschhauser, 2020 #194] and sarcoma [Gounder, 2020 #195], and shows therapeutic efficacy. Therefore, EZH2 inhibitors might be clinically available to confirm the anti-tumor action against melanomas.

## 17. Inhibition of Bromodomain and Extra-Terminal Domain (BET) Downregulates PD-L1

BET proteins present two tandem bromodomains (BD1 and BD2), an extra-terminal domain (ET), and a C-terminal domain (CTD), and they mainly recognize and connect to the acetylated lysine of histone 4 [121]. BET proteins contribute to the recruitment of other epigenetic proteins for transcriptional activation. A BET bromodomain inhibitor, JQ1, decreases PD-L1 expression and tumor progression in prostate cancer models [122].

A BET inhibitor, iBET726, suppresses PD-L1 in addition to MHC class I and cancels the immunotherapy effect in a melanoma model [65], suggesting a possible disadvantage of the BET inhibitor in an anti-tumor immune response to melanoma. Therefore, a stimulant for BET-targeted treatment may be expected to enhance the anti-tumor effect of PD-1/PD-L1-targeted treatment against melanoma.

## 18. DNA Hypermethylation in TNFRSF9 during Anti-PD-1 Treatment

The T-cell costimulatory receptor TNFRSF9 is known as a novel target for immunotherapy [123,124,125]. TNFRSF9 is expressed on the surface of certain immune cells such as activated T cells and interacts with its ligand in activated antigen-presenting cells, leading to the activation of immunity against cancer [126]. DNA hypermethylation at the TNFRSF9 promoter site is associated with a reduced TNFRSF9 mRNA expression and unfavorable clinical behavior during anti-PD-1 antibody treatment [127], suggesting that the tumor might drive another advantageous strategy against the anti-tumor immune response during anti-PD-1 antibody treatment.

## 19. DNA Hypermethylation in RAD51B Upregulates PD-L1

RAD51B is essential for DNA repair and regulates various malignant proliferation processes [128]. The methylation of RAD51B is correlated with patient outcome in non-small-cell lung cancer. RAD51B methylation levels were positively related to high PD-L1 expression [129]. A highly upregulated PD-L1 is related to a favorable clinical response, and patients with PD-1 blockade efficacy had higher RAD51B methylation levels [129]. Therefore, RAD51B methylation stimulants are expected to obtain therapeutic efficacy in anti-PD-1/PD-L1 antibody treatment.

## 20. p300/CBP Activates Anti-Tumor Immunity Mediated by MHC Class I Upregulation in Tumors

p300 and CPB modulate gene transcription through direct lysine acetyltransferase catalytic activity for acetylation of histone or non-histone proteins [130,131]. They are essential during the development of normal hematopoietic stem cells [132]. Recently, the inactivation of CBP and p300 has been reported in malignancies [133]. The suppression of MHC class I is one of the strategies for the immune escape phenomenon in cancer cells, leading to lower efficacy for immunotherapy. p300/CBP positively regulates MHC-I expression in the tumor cells [134]. Since the efficacy of the p300/CBP activator CTPB was investigated in an in vitro experiment [135], this stimulant might obtain an anti-tumor immune response to melanoma.

## 21. KDM4A Suppresses Anti-Tumor Immune Response

KDM4A is a histone H3 lysine 9 trimethylation demethylase, and it plays a vital role in malignancies [136,137,138,139]. KDM4A inhibition promoted the anti-tumor immune response against squamous cell carcinoma [140]. The inhibition of KDM4A activates the formation of liquid-like HP1γ puncta on heterochromatin and causes DNA replication, which promotes intrinsic cGAS-STING signaling in the tumor cells [140]. STING signaling is a highlighted immune regulator and enhances type I IFN production, leading to enhanced anti-tumor immune response. Therefore, STING-targeted therapy is expected to be useful in cancer immunotherapy in combination with anti-PD-1 antibody treatment. The combination therapy of KDM4A inhibition and anti-PD-1 antibody suppresses tumor growth mediated by activation of CD8^+^ T cells [140]. STING stimulants are currently expected as immunotherapies [40]. The STING pathway also enhances autophagy as an anti-tumor effect [141], in addition to DNA damage response to tumor cells [142] and MHC class I expression in tumor and immune cells [143].

## 22. USP7 Upregulates PD-L1 in Tumors

Histone ubiquitination is one of the epigenetic histone modifications, and histones are typically ubiquitinated on lysine residues contained within histone tails, leading to transcriptional silencing [144]. Ubiquitin is removed from histones by a deubiquitinating enzyme, leading to transcriptional activation. USP7 is a deubiquitinase that regulates many diverse cellular processes, including tumor suppression. PD-L1 expression is positively correlated with USP7 expression in gastric cancer [145]. USP7 suppression impairs PD-L1/PD-1 interaction and enhances the cytotoxic reaction of T cells and the anti-tumor immune response [145].

Although there has been no investigation into the effect of chemical USP7 inhibitors in melanoma cells, the USP7 inhibitors FT827 and FT671 showed high potency for suppressing USP7 activity in breast cancer cells [146]. These inhibitors are expected to advance to further clinical trials for the treatment of melanoma.

## 23. Summary of Epigenetic Alteration Influence in Immunotherapy

Table 1 summarizes the influence of epigenetic changes in immunotherapy. DNA hypermethylation impairs the anti-tumor immune response. DNA hypomethylation has become a strategy for malignant melanoma treatment. On the other hand, DNA hypomethylation enhances PD-L1/PD-L2 expression and reduces the cytotoxic reaction against malignant tumors. Therefore, a DNA hypomethylation agent, azacytidine, is useful to obtain additional therapeutic efficacy in anti-PD-1/PD-L1 antibody treatment.

Histone acetylation mediated by HDAC inhibitors is expected to obtain additional therapeutic efficacy in combination with an anti-PD-1/PD-L1 antibody. Histone acetylation activates cytotoxic immune reactions to tumors and enhances MHC class I/class II expression in tumors. Since PD-L1 is also upregulated by HDAC inhibitor, anti-PD-1/PD-L1 antibody treatment is expected to cover this advantage.

In contrast, histone methylation reduces PD-1 expression and enhances the cytotoxic reaction to tumors. KDM4A inhibitor accelerates histone methylation and activates cytotoxic immune reactions. EZH2 also enhances histone methylation and downregulates PD-1 expression.

Histone ubiquitination mediated by USP7 upregulates PD-L1 expression. Therefore, a USP7-targeted inhibitor is expected as an additional combination therapy with an anti-PD-1/PD-L1 antibody treatment.

Table 2 summarizes the currently available pharmacological epigenetic modifiers that could be used in the future. HDAC inhibitors are currently being developed as a treatment option for clinical patients and could be used practically as a clinical application in the future. The clinical trial of the HDAC6 inhibitor nexturastat A has not yet been conducted. Clinical trials of the DNTM inhibitor azacytidine and the EZH2 inhibitors tazemetostat and GSK126 are currently being conducted with respect to various malignancies; therefore, these agents are also expected to confirm the efficacy of the anti-tumor immune response in combination with anti-PD-1/PD-L1 antibody treatment.

## 24. Conclusions

We summarized the current development of epigenetic-modification-mediated immunotherapy. Since there is a lack of knowledge regarding melanoma, these findings were obtained from studies on other malignancies, but similar epigenetic influences are expected with regard to cutaneous malignant melanoma. It is also desirable to identify novel candidate molecules for immunotherapy, since the tumor side establishes a resistance to these immunotherapies mediated by epigenetic modification mechanisms, to alter the gene expression by acquired environmental conditioning. Therefore, epigenetics-targeted therapy combinations might overcome the limitation of immunotherapy alone. However, systemic epigenetic modifier application has an influence on both tumor and healthy cells. Therefore, adverse reactions to the influence of these epigenetic modifiers must to be examined.

## Figures and Tables

**Table 1:** Epigenetic modifications and effects.

Epigenetic Modification	Target Enzyme or Gene	Effects
DNA hypomethylation	DNA hypomethylation agent Azacytidine	PD-L1↓ PD-L2↓
DNA hypomethylation	DNMT inhibitor	Cytotoxic immune reaction↑
Histone acetylation	HDAC inhibitor	Cytotoxic reaction↑ PD-L1↑ MHC class I↑ MHC class II↑
Histone demethylation	LSD1	Cytotoxic reaction↓ CD8↓ CCL5, CXCL9, CXCL10↓ PD-L1↓
DNA hypermethylation	PRF1	PD-L1↑
Histone methylation	EZH2	PD-1↓
Histone deacetylation	BET inhibitor JQ1	PD-L1↓ MHC class I↓
DNA hypermethylation	RAD51B	PD-L1↓
Histone acetylation	p300/CBP	MHC class I↑
Histone methylation	KDM4A inhibition	Cytotoxic reaction↑
Histone ubiquitination	USP7	PD-L1↑

**Table 2:** The possible therapeutic epigenetic modifiers confirmed in this review.

Epigenetic Targets	Agents
HDAC inhibitor	Panobinostat Entinostat Mocetinostat Vorinostat Chidamide
HDAC6 inhibitor	Nexturastat A
DNMT inhibitor	Azacytidine
EZH2 inhibitor	Tazemetostat GSK126

## Data Availability

Not applicable.
